# Transfer of clinical debriefing from simulation to practice: exploring the barriers and enablers

**DOI:** 10.1186/s41077-025-00405-8

**Published:** 2026-01-12

**Authors:** Charlotte Jane Dewdney, Stephen Richard Waite, Katherine Ralston, Emma Claire Phillips, Edward Mellanby, Victoria Ruth Tallentire

**Affiliations:** 1 Postgraduate Education Centre (PEC), Royal Infirmary of Edinburgh, 51 Little France Crescent, Old Dalkeith Road, Edinburgh, EH16 4SA UK; 2 Medicine for the Elderly Department, St John’s Hospital, Howden Road West, Howden, Livingston, EH54 6PP UK; 3 NHS Education for Scotland, 102 West Port, EH3 9DN Edinburgh, UK

**Keywords:** Clinical debriefing, Simulation, Training transfer, Work environment

## Abstract

**Background:**

Clinical debriefing (CD) positively impacts individuals, teams and systems and has been shown to improve patient outcomes and staff wellbeing. Although there is a growing evidence base supporting CD, it has not been routinely adopted by many healthcare organisations. Despite the work environment being an important component of transfer of learning, there has been minimal focus on how it influences implementation and maintenance of CD in practice. The overall aim of this study was to explore the work environment barriers and enablers influencing the transfer of clinical debriefing skills from simulation to clinical practice.

**Methods:**

Following ethical approval, medical registrars who had participated in a simulation course involving a within-scenario CD were invited to participate in semi-structured interviews. These utilised Burke and Hutchins’ evaluation model as the initial conceptual framework and took place at least two months post-course. Interviews explored participants’ experiences of transferring learning related to CD from simulation to the clinical workplace, and were transcribed verbatim and dual coded using template analysis.

**Results:**

Fifteen medical registrars participated in interviews between January and May 2025. The work environment influences from Burke and Hutchins’ evaluation model resonated as important factors affecting adoption of CD. With the addition of subthemes generated inductively from the data, the model provided a framework for identification and articulation of the barriers and enablers to CD in the workplace. The most striking finding was participants’ sense of personal responsibility to engage with CD. In addition, participants identified the requirement for cultural change to enable CD.

**Conclusions:**

Work environment influences represent both barriers and enablers of CD in relation to the transfer of learning from simulation to clinical practice. Personal responsibility and workplace culture are important drivers of CD, and attention should be paid to the influence of both constructs in this context. Recommendations for practice, based on our findings, are designed to enable educators and organisations to promote the adoption of CD in their own settings. This will help to bridge the gap and make CD the norm, not the exception.

**Supplementary Information:**

The online version contains supplementary material available at 10.1186/s41077-025-00405-8.

## Background

There is a growing body of evidence advocating for clinical debriefing (CD) to positively impact individuals, teams and systems through improved patient outcomes and staff wellbeing [1–3]. A CD is a group learning conversation, which attempts to bridge the gap between the experience of a clinical event and making sense of it [4]. A CD may be undertaken after specific clinical events or as a part of routine practice; it represents an opportunity for all members of a clinical team to share their experiences and learn from them [1]. Clinical debriefing contributes to improved patient outcomes, such as enhanced cardiopulmonary resuscitation survival, as well as better identification of clinical and systems-level errors [5, 6]. It also supports staff wellbeing by fostering psychological safety and team cohesion [7]. The evidence is compelling: clinical debriefing works [8, 9]. Despite this, CD has not been consistently adopted across healthcare settings [10–12]. Multiple barriers to its implementation have been described, including lack of time, deficiency of skilled facilitators and limited understanding of its utility [7, 13–15]. To date, efforts to address this gap have primarily focussed on developing debriefing tools and training facilitators [16, 17]. However, the potential role of simulation to support the routine uptake of CD remains unexplored.

Simulation-based education (SBE) offers a powerful platform for experiential learning, reflection, and deliberate practice [18, 19]. Within simulation, a debriefing is defined as a guided conversation among participants that aims to explore and reflect on the experience of a simulated scenario by drawing upon experiential learning theory [20]. CD has many parallels with debriefing within simulation, the key difference being that a CD is in response to a real-life scenario. Despite the similarities and crossover, opportunities to rehearse clinical debriefing itself - particularly in situ or as a transferable workplace skill - are rarely integrated into simulation curricula. While simulation has been successfully used to train teamwork and communication skills [21, 22], its role in preparing healthcare professionals to lead or participate in clinical debriefings remains unclear.

Simulation offers a promising opportunity to facilitate learning about CD. Enabling participants to perform a simulated CD, followed by a faculty lead post-scenario discussion should allow participants to develop a deeper understanding of CD and how they might apply this learning within their own clinical settings. However, to date we do not understand that factors that influence the transfer of CD skills from simulation to clinical practice. A better understanding will support the development of more effective SBE activities, thereby bridging the theory-practice gap and enabling transfer of learning to real-life engagement in CD in the workplace.

### Conceptual Framework

Transfer of learning is an enduring problem in medical education [23]. Simulation is no exception and the issue of transfer of learning from the simulation environment to ‘real-life’ situations is complex and challenging [24]. A pivotal paper by Baldwin and Ford (1988) identified three main factors that influence transfer of training: trainee characteristics, training design and the work environment [25]. Burke and Hutchins [26] expanded on this work with an integrative review of training transfer exploring the three transfer factors in Baldwin and Fords’ conceptual model in greater detail. They found that *trainee characteristics* influencing transfer include motivation and perceived utility; both highly relevant to CD. *Training design* constructs include learning objectives and technological support, which are pertinent to the underlying design of a simulation scenario. The final consideration is the *work environment* which includes: transfer climate; supervisor and peer support; accountability; opportunity to perform; and strategic link.

**Fig. 1:**
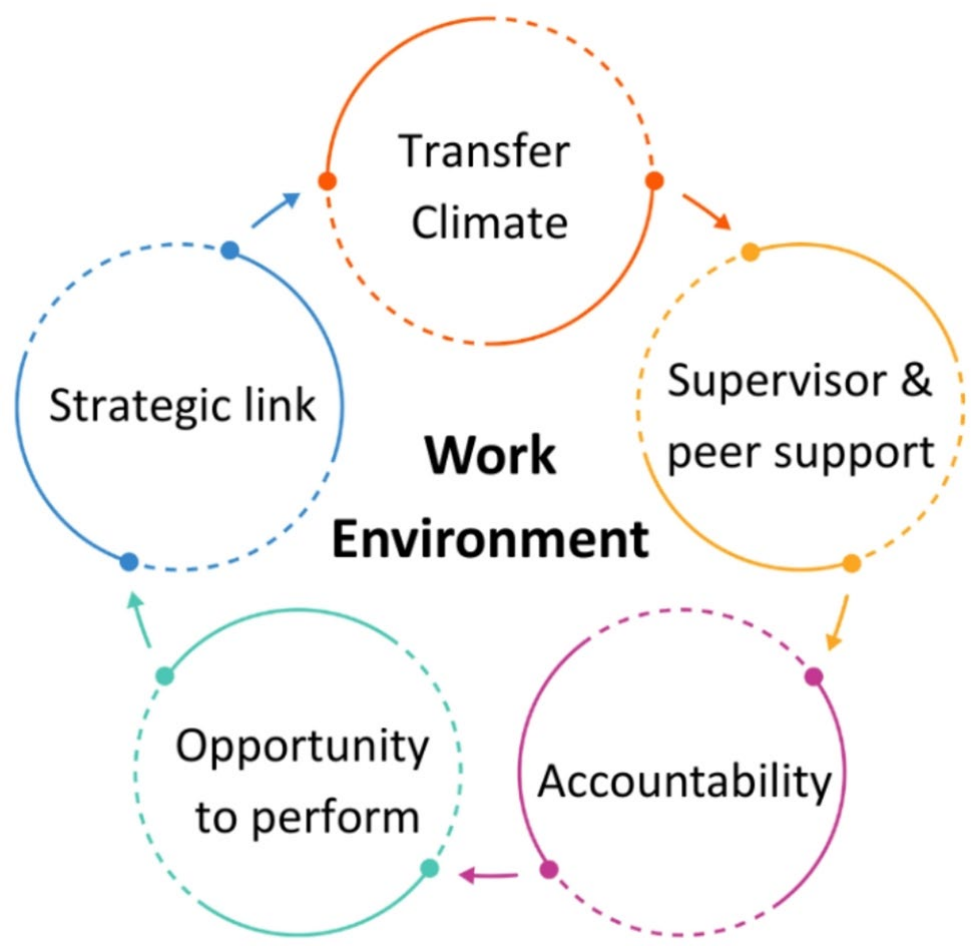
Illustration of the work environment influences adapted from Burke & Hutchins’ evaluation model [26]

While previous studies have considered some aspects of simulation transfer [27, 28], none have focused explicitly on the work environment factors that influence the uptake and sustainability of CD in practice. These factors are vital to understand if CD is to become a routine, supported practice rather than a sporadic initiative. To address this gap, we adopted Burke and Hutchins’ work environment influences as our conceptual framework (Fig. 1). This allowed us to explore, in a systematic and theory-informed way, the contextual barriers and enablers that affect whether simulation-based learning in CD translates into real-world implementation.

### Aim

The overall aim of this study was to explore the work environment barriers and enablers influencing the transfer of clinical debriefing skills from simulation to clinical practice.

## Methods

### Study participants

In the United Kingdom (UK), medical registrars are senior doctors who are training to become consultants. Following completion of medical school, doctors undertake two years of Foundation Training. Those wishing to pursue a career in medicine typically enter stage 1 of Internal Medicine Training, a three-year programme covering a range of medical specialties. Medical trainees can then decide to continue with stage 2 of Internal Medicine Training where they typically dual train in internal medicine and another medical specialty. It is at this point they are commonly referred to as ‘medical registrars’. The medical registrar role is held with both reverence and fear [29]. Often described as the ‘workhorses’ of the National Health Service (NHS), medical registrars are expected to demonstrate exceptional leadership skills, as the cardiac arrest and medical emergency team lead [30]. Given that CDs are often prompted by unanticipated clinical events that involve high acuity [31] medical registrars significantly influence the implementation of CD in practice.

### Simulation course

Between November 2024 and April 2025, a ‘Simulation for Medical Registrars’ course was delivered to 32 medical registrars, in groups of up to seven. All medical registrars within South-East Scotland were invited to participate. The course was a one-day immersive simulation course with six scenarios focusing on behavioural skills and human factors. Intended learning outcomes were modelled around the General Medical Council (GMC) Generic Professional Capabilities [32]. The course was piloted three times in 2023, with faculty meetings and participant feedback guiding iteration after each pilot. Within the course, one scenario involved a simulated obstetric medical emergency, following which participants led a within-scenario CD using the STOP5 debriefing tool (Additional File 1) [33]. STOP5 is a structured tool to facilitate short (five-minute) clinical debriefings [34], which requires the participants to *s*ummarise the case, *t*hings that went well, *o*pportunities to improve, and *p*oints to action. STOP5 was chosen as it is simple, provides an easy structure, and is designed to facilitate a brief CD with any team member as a facilitator [17]. The 15-minute scenario (including the within-scenario CD) was followed by a 45-minute facilitator-led debriefing, during which participants were given the opportunity to discuss and analyse the within-scenario CD.

The course was primarily developed to improve patient safety and quality of care by encouraging participants to translate lessons learned from the simulation back into clinical practice. Its design was informed by Kolb’s experiential learning theory [35], which consists of four phases. (a) A within scenario CD provided participants with a *concrete experience*, (b) this was followed by *reflective observation* facilitated by the post-scenario discussion. (c) Following the course, participants were able to consider and reflect on the simulation as part of *abstract conceptualisation*, (d) finally participants were encouraged to perform CD in practice through *active experimentation*.

### Data collection and analysis

Having provided consent for subsequent contact at the simulation course, all participants were contacted via email two to three months later, and invited to take part in this research study. Semi-structured interviews were chosen as the method of data collection to acquire in-depth information with a degree of flexibility in the questions. Interviews were conducted by CJD via Microsoft Teams between January and May 2025, and were audio-recorded and transcribed verbatim. The interview schedule (Additional File 2) was modelled around Burke and Hutchins’ evaluation model to allow deeper exploration of themes relevant to transfer of learning [26]. Interview transcripts were dual coded using template analysis [36], with Burke and Hutchins’ work environment influences informing the initial coding template.

Analysis of the early interviews commenced in parallel with on-going data collection to allow deeper exploration of themes. The researchers met frequently during the data collection and analysis phases to discuss each category of the framework in detail. In keeping with template analysis, a priori codes were refined iteratively alongside ongoing data collection and analysis. Interviews continued until the sample size was deemed to have sufficient information power to address the research question. This judgement involved consideration of the study’s narrow aim, specificity of the participant group (medical registrars), use of a conceptual framework, and quality of the interview data co-constructed between interviewer and participant [37]. Member checking was performed whereby participants reviewed their transcripts and were given the opportunity to comment on synthesised findings from the study.

### Philosophical orientation and reflexivity

This study employs a constructivist approach to explore the work environment barriers and enablers to CD. Constructive ontology holds the view that there are multiple realities to uncover and that each of these realities arises from the ‘construction’ of understanding, based on the participant’s context, previous experience, attitudes and beliefs [38]. Using a constructivist approach, authors’ subjective perspectives are fundamentally intertwined with the research process [39]. These ‘inner conversations’ are a product of researchers’ prior experiences and can influence the study’s design and findings [40]. The research team are from a variety of clinical backgrounds including anaesthesia (SRW, ECP, EM), general medicine (CJD, VRT) and geriatrics (KR) and collectively have significant expertise in simulation-based education including facilitation (all), research (KR, ECP, EM, VRT) and faculty development (KR, ECP, EM). We recognise that all authors are advocates of CD and have a personal interest in exploring the barriers to CD. In line with constructivist epistemology, we attempted to harness these perspectives in a reflexive manner, emphasising the co-construction of ideas by the lead researcher, co-researchers and participants [41].

### Ethical approval

This study received ethical approval from the NHS Education for Scotland ethics review board (reference number NES/Res/46/22/Med). Participants gave written consent for data collection and the publication of anonymised results. They were free to leave the study at any time, up to the point of data analysis.

## Results

Fifteen participants took part in online interviews between January and May 2025. Interviews lasted between 14 and 41 minutes (mean 22 minutes). Seven participants identified as male and eight as female. They were aged between 30 and 45 years.

**Fig. 2:**
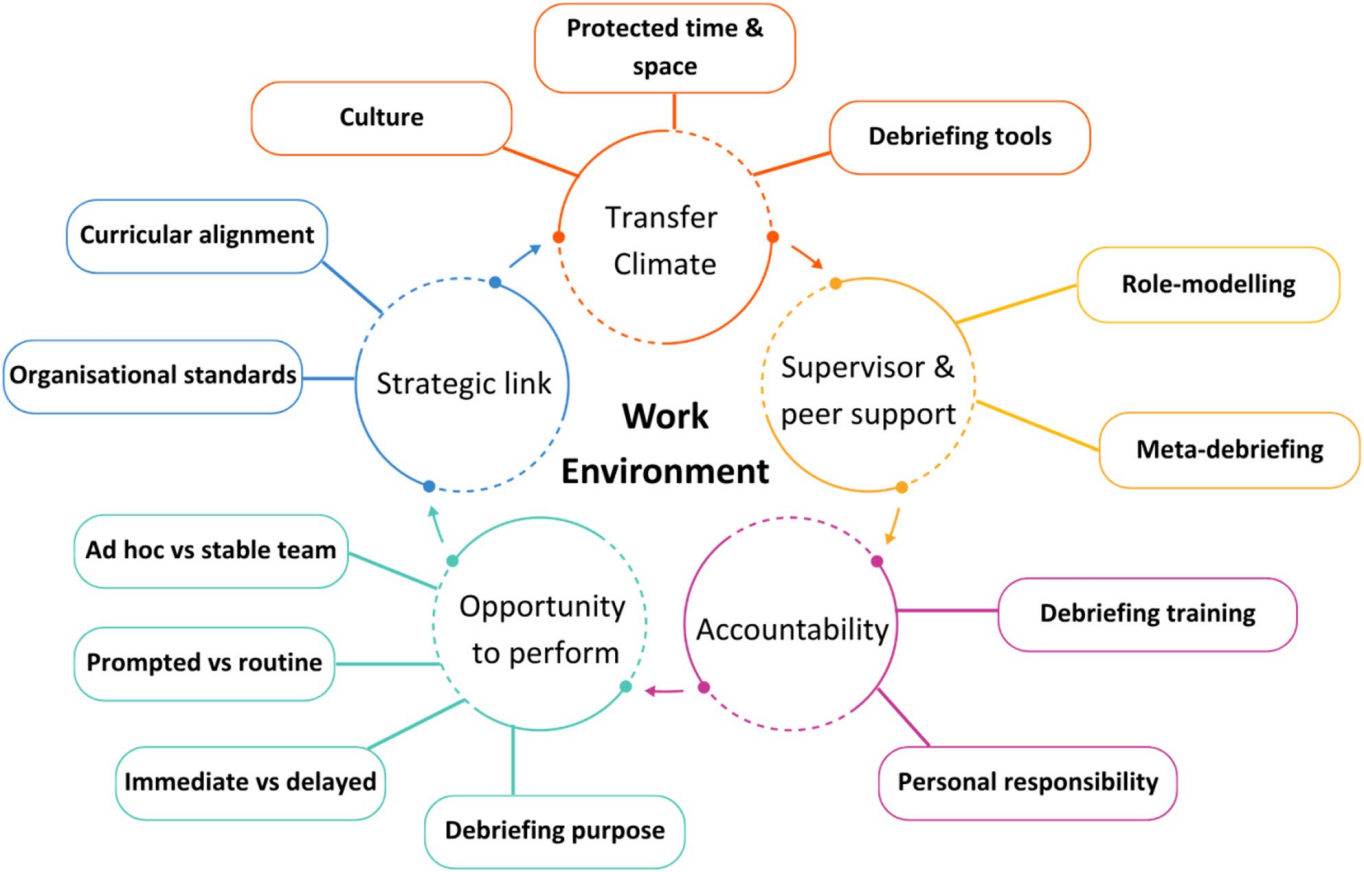
Illustration of the work environment influences from Burke & Hutchins’ evaluation model [26] with the addition of subthemes generated inductively from the data

The work environment influences from Burke and Hutchins’ evaluation model [26] resonated with participants as important factors affecting adoption of CD in practice. With the addition of subthemes generated inductively from the data (Fig. 2), the framework revealed both barriers and enablers to CD as illustrated by example quotes (Table 1).

**Table 1:** Work environment barriers and enablers to CD, based on by Burke and Hutchins’ evaluation model [26] with subthemes shown in italics and example quotes

**Themes**	**Subthemes**	
	Barriers	Enablers
Transfer climate ‘The overall workplace conditions that either encourage or inhibit the application of CD’	*Culture*	
	“There's not necessarily a culture of debriefing; it's not something that we talk about at huddles” (P4)	“…the more people that are doing it, I think culture grows culture” (P8)
	*Protected time and space*	
	“The main barrier is probably time and also an appropriate location” (P2)	“...there's nowhere that doesn't have a doctor's room or some sort of space” (P8)
	*Debriefing tools*	
	“I find them difficult because I feel like I don't have a structure” (P1)	“Definitely having a tool is really helpful because it doesn't make it this ambiguous, scary thing” (P2)
Supervisor & peer support ‘The support registrars receive to use their new skills and knowledge’	*Role-modelling*	
	“I've never really had a lot of experience of people debriefing with me when I was junior” (P9)	“I think the resus officers are quite helpful…because they're another team lead, they're quite supportive” (P5)
	*Meta-debriefing*	
	“How do we debrief the med regs themselves? Because I think if we've been debriefing everybody else, it's sometimes really difficult to know who we can speak to about events that have been quite tricky” (P8)	“If you think of a debriefing as like a real team event, then I guess you are debriefing yourself” (P11)
Accountability ‘The degree to which the organisation expects registrars to use trained knowledge on the job and holds them responsible for doing so’	*Debriefing training*	
	“Not everyone gets education and training about debriefing and how to do it or facilitate it and then they don’t know the importance of debriefing” (P3)	“I now have had some teaching in it, and I kind of know roughly what I'm doing with it and can put it into practice” (P11)
	*Personal responsibility*	
	[when reflecting on missed debriefing opportunities] “People said to me, this must be the only job that you go and see an emergency or see someone die and then just get back to work. And I remember being like, ‘yeah, it is.’” (P10)	“You don't want to be the person not doing the thing that everybody else is doing. So yeah, I guess there is a personal responsibility as well” (P11)
Opportunity to perform ‘Whether registrars are provided with opportunities to use new learning in their work setting’	*Debriefing purpose*	
	“Most of debriefings I’ve been in are because things have gone badly and it has been to check how everyone is feeling afterwards” (P5)	“Having to think about the purpose of debriefing has made me feel a bit more confident about doing it” (P11)
	*Immediate vs delayed*	
	“I think events are fresh in your mind and the right people are there. Whenever you try and do something later on, inevitably people can't make it.” (P11)	“A cold [delayed] debrief allows you to take some of that emotion out of it and analyse it and go through it in much more of a structured way” (P12)
	*Prompted vs routine*	
	“Normally, if it was a bad situation - if something went really well, normally that wouldn't trigger thinking about debriefing” (P4)	“But I guess if it is commonplace, and people know that this is not that specific case, and everyone's doing it in a very objective way, then that might actually make things better” (P9)
	*Ad hoc vs stable team*	
	“You have different nurses almost every night, and you have people that are covering random clinical areas that you never see again. I think it [debriefing] is a challenge the less often you see a team” (P8)	“[when you work in an ad hoc team] you feel more comfortable sharing opinions in a more sort of anonymised setting” (P14)
Strategic link ‘The organisation’s goals and strategies’	*Curricular alignment*	
	“We don’t really talk about it in specialty teaching” (P5)	“I think if it's something you had to get signed off, or some kind of education about for your portfolio, that would be quite helpful for everyone” (P13)
	*Organisational standards*	
	“I think we can easily slip into service mode, being more of a service provision and just task oriented” (P3)	“Adding it to an arrest trolley or one of the forms for arrests, might help to encourage people to do it” (P12)

### Transfer climate

Transfer climate encompasses the overall workplace conditions that either encourage or hinder the application of clinical debriefing in practice. We identified culture, protected time and space, and debriefing tools as salient subthemes. Participants emphasised the importance of culture; there was hope that introducing CD at an earlier career stage would encourage cultural change. Education and training were deemed important in instigating cultural change: “you have to work through raising awareness of it, educating and creating that culture.”(P15). A frequently recognised barrier to CD was time, and many participants felt that a high clinical workload prohibited CD from happening. Similarly, finding a suitable space could be challenging: “we don’t really have a lot of locations that are free for a debrief[ing]. Sometimes you find a spare room, sometimes you find a quiet corridor.”(P2).

Conversely, CD-enabling actions included setting expectations at the start of a shift and challenging the misconception that CD is overly time-consuming: “Set that standard at the morning meeting, ‘we are going to debrief after this, and it will only take 5–10 minutes.’”(P5). The final subtheme within transfer climate related to use of debriefing tools. The use of a debriefing tool, as introduced in the simulation course, was a strong enabler: “you’ve got a million different things that you need to remember on night shifts; unless I have a tool or a way of structuring something in my head, it just doesn’t happen.”(P11).

### Supervisor and peer support

Supervisor and peer support includes the encouragement that medical registrars receive to use their clinical debriefing skills in practice. Role-modelling from colleagues was considered an important subtheme: “I think the supervisor or group of peers definitely have a big impact; what your peers do, and what the consultants expect you to do, will definitely impact that a lot.”(P4). However, it was noted that debriefing support could be lacking within some teams: “sometimes people don’t want to chat through things that they’ve not been directly involved with. It’s consultant dependent.”(P8). In contrast, some participants recounted positive experiences, particularly relating to acceptance and uptake of debriefing: “I felt that whenever you do a debrief[ing], most of the team members would receive it well.”(P7). Certain members of the team, such as the resuscitation officers, were identified as being particularly important in positively influencing uptake of CD

In relation to CD, there was a palpable sense of isolation amongst the participants: “we feel quite alone in the sense that sometimes we don’t get debrief[ing]s with the consultants the next day.”(P2). Indeed many of the participants remarked that they didn’t discuss CD or have an appreciation of whether their colleagues were engaging with CD. Within simulation, meta-debriefing is recognised as a vital component of enhancing debriefing skills. In this study, the subtheme of meta-debriefing referred to debriefing the participants themselves, after they had performed a CD. In keeping with a sense of isolation, the importance, and deficiency, of meta-debriefing was mentioned by several participants: “there is this expectation that you will just sort of manage it, and get on with it, when actually, you might be the person with the most to deal with… and that might feel quite heavy on you.”(P14).

### Accountability

Accountability is the degree to which the organisation expects medical registrars to engage with clinical debriefing and holds them responsible for doing so. Participants emphasised the importance of debriefing training, which formed a subtheme within the theme of accountability. It was clear that participants’ experience of CD varied considerably across the cohort: “I don't think I had any teaching or training on it.”(P11). Participants reflected that after the simulation course they felt empowered to put debriefing into practice in their own setting. One participant remarked: “it was a big wake-up call thinking, oh my goodness, as a registrar I’ve not actually done that… since then I've tried to make an effort to debrief.”(P2).

Regarding the subtheme of personal responsibility, some participants felt that any member of a team should feel able to initiate and lead a debriefing. However, they recognised that this wasn’t the case in practice: “the responsibility mainly lies with the registrar at the moment.”(P9). Exploring the rationale behind this, there was consensus that the team lead – often the medical registrar – carries the responsibility to debrief. A sense of personal responsibility was a powerful influencer for many of the participants and for some it evoked emotive reflections: “People said to me, this must be the only job that you go and see an emergency or see someone die and then just get back to work. And I remember being like, ‘yeah, it is.’”(P10). Participants reflected on their perception of responsibility and how they might leverage it to enable debriefing to happen, both amongst their peers and by inspiring the future generation: “I just think we need to speak about it more. I think that, as registrars, if we start debriefing with our juniors, hopefully they’ll pick up on that and when they do get to a similar position, hopefully they’ll carry that on.”(P2).

### Opportunity to perform

This theme explores whether registrars are provided with opportunities to perform clinical debriefing in their setting. Understanding debriefing purpose was a subtheme, and some felt that it was essential to debrief after certain clinical events, in order to process emotions and continue working safely and effectively during a shift: “taking time to recognise that something's happened and not go straight back to work.”(P12). The timing of the debriefing was also important. From a practical perspective, participants agreed that debriefings were more likely to happen immediately after an event rather than if they were delayed to a later time. However, immediate debriefings have their challenges. One participant noted: “you just have to accept that some people don't want to talk about things at the time.”(P1).

Participants agreed that the typical situation prompting a debriefing is a cardiac arrest. However participants also reflected that debriefing does not have to be limited to this circumstance, and could be performed as part of routine work: “you should do it regularly… irrespective of what you think has been done well or poorly.”(P9). This may then help to remove any negative connotations associated with debriefing, as some participants reported that it is only performed when things go wrong. Finally, team dynamics were perceived to be significant. Some participants felt that, given the nature of hospital shifts, working with ad hoc teams (i.e. short-lived teams where participants often vary) could be a barrier to debriefing: “you don't know people as well [when working in an ad hoc team]. You're not as connected to them. You don't know their personality, their emotions, whether it's helpful to talk about these things. Some people are receptive to it. Some people aren’t.”(P13). This may have implications for organisations and educators, emphasising the importance of continuity, while simultaneously highlighting the challenge of building trust and cohesion in fluid teams.

### Strategic link

Strategic link refers to the organisational goals and strategies aligned with clinical debriefing. Participants reflected that one of the most prevalent barriers to CD is a lack of organisational guidance and protocols. Some participants felt that their departments prioritised other tasks over debriefing, or that it was not an activity promoted in their area of work: “I don't think it is highlighted as something that should be done.”(P5). Participants agreed that to increase uptake, debriefing should become part of the medical registrars’ curriculum. One participant commented: “non-technical skills, such as clinical debriefing, have lagged behind clinical knowledge but they're certainly becoming part of what's expected, and therefore maybe they should be part of the curriculum and assessed in the same way as other parts of the curriculum.”(P11). Medical registrars have an online professional portfolio that is assessed on an annual basis to determine progression. One suggestion was for CD to form part of this assessment. It was also proposed by participants that clinical debriefing could become standard practice within the organisation, through inclusion in medical emergency and cardiac arrest protocols.

## Discussion

Clinical debriefing is a powerful tool to improve patient outcomes and staff wellbeing [2, 5], yet its potential remains unrealised within many healthcare organisations. This study used the work environment influences from Burke and Hutchins’ evaluation model [26] as a framework to explore the transfer of learning relating to CD from simulation to clinical practice. Many participants had not considered or engaged CD before participating in the simulation. Enabling participants to experience and discuss CD within simulation, followed by interviews exploring its subsequent application in clinical settings, allowed us to elucidate the contextual barriers and enablers to CD. Frequently cited barriers include lack of time and space, and unclear organisational goals, whereas key enablers include debriefing training and use of debriefing tools. Our hope is that organisations and educators will be able to utilise this nuanced understanding of CD in practice to enhance uptake and capitalise on the benefits.

**Fig. 3:**
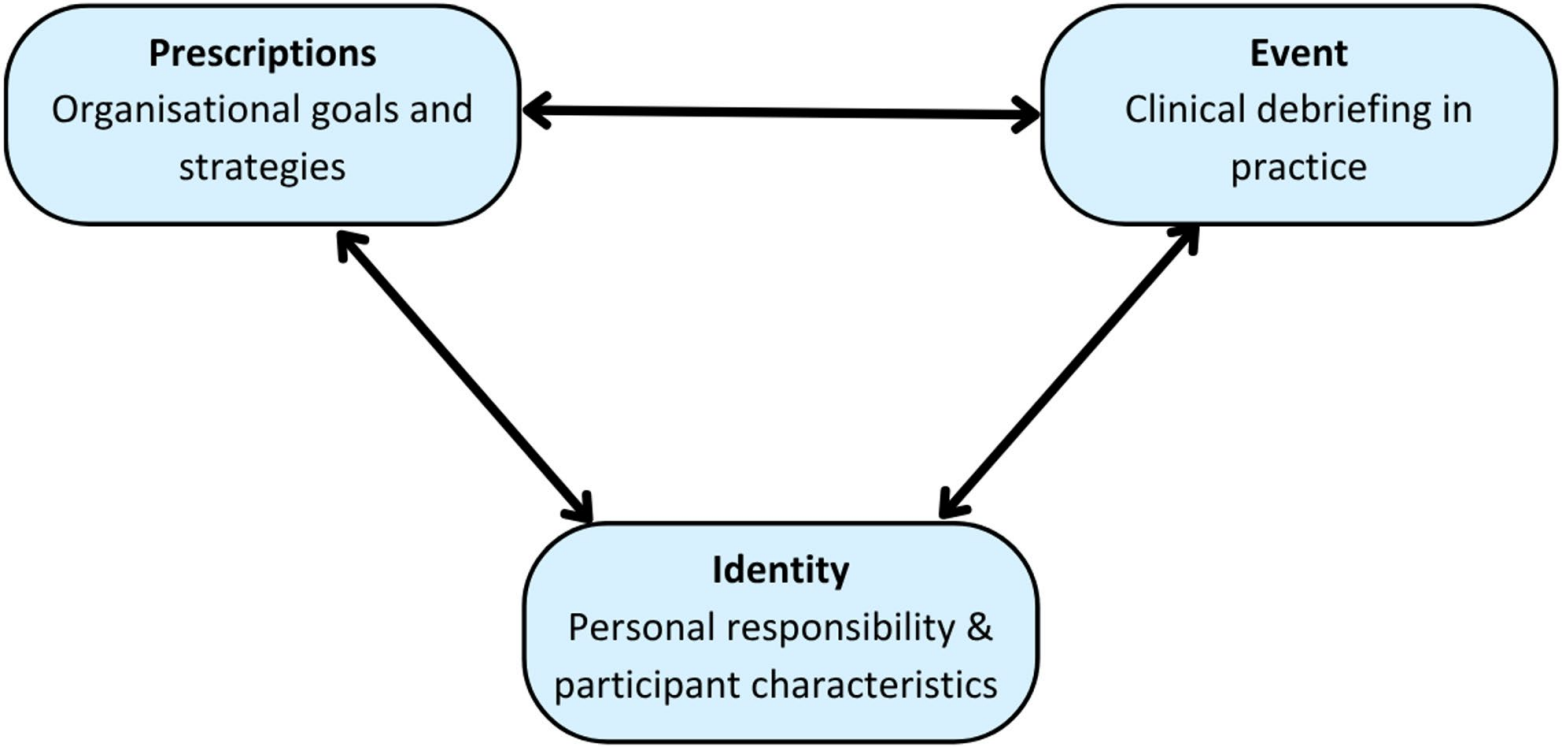
The responsibility triangle holding participants accountable for CD in practice, adapted from Schlenker et al. (1994) [42]

The finding from this study that we found the most striking was participants’ sense of personal responsibility to perform CD. This study has identified a range of work environment barriers, which may at times seem insurmountable. However, those with the strongest sense of personal responsibility felt that they could overcome any work environment barrier to CD and ensure its implementation. Given its significance, the question we must address is how can we foster a sense of personal responsibility amongst clinicians to make CD a reality? The original transfer model includes trainee characteristics, training design and the work environment. Arguably, nomenclature dictates that personal responsibility is inherent to trainees’ characteristics rather than the work environment. We found that they are interdependent: cultivating a work environment supportive of CD heavily influences personal responsibility. Of relevance is Schlenker *et al.’s.* (1994) accountability theory [42], which describes the ‘responsibility triangle’ (Fig. 3). In the context of this study, the responsibility triangle holds participants accountable for CD in practice. It includes prescriptions (professional standards governed by the healthcare organisation), identity (participants’ personal attributes and their sense of responsibility) and the event (CD in practice). The links between these three variables form the adhesive glue that binds participants to certain situations and holds them accountable for debriefing in clinical practice (Fig. 3).

Personal sense of responsibility can have important implications for motivation [43] and can affect the feeling of responsibility to fulfil professional requirements [44]. Many healthcare professionals are registered with a governing body and so are subject to professional standards, but there is a need to better understand how this translates to personal responsibility. From our study, personal responsibility to perform CD was multifactorial and depended on participants’ attributes, the training they had received, and characteristics of the organisation in which they worked.

All the themes and subthemes identified in this study are interconnected, and it is important to consider these links when interpreting the data. For example, transfer climate includes the subtheme of culture, and participants identified the requirement for cultural change to enable CD. Nilsen et al. (2020) explored the characteristics of successful change within healthcare [45]. They found that change is more likely to succeed when professionals have the opportunity to both influence and recognise the value of the change, including perceiving its benefit for patients. Simulation is a powerful tool to enable participants to understand the value of CD and its potential for generating positive clinical outcomes. As such, as others have shown [46–48], it has the potential to promote cultural change both in terms of improving patient outcomes and enhancing teamwork. However, individual participants’ learning from simulation can only go so far in influencing change. For significant change to occur, and for CD to be sustainable, strategic link is important: CD needs to be part of an organisation’s goals and strategy.

In this study, participants were unaware of specific organisational goals and strategies in relation to CD. They did not feel that CD was promoted by their workplace as a leadership skill they should demonstrate and develop. Indeed, one of the criticisms of the medical registrar curriculum is a deficiency in leadership training, and the landmark Shape of Training review [49] put an emphasis on increasing such opportunities. Simulation can be a vehicle to foster leadership skills [50], and the United Kingdom’s postgraduate Internal Medicine curriculum now includes a mandatory requirement for simulation training [51]. Perhaps now is the time to catalyse this change: introducing clinical debriefing as part of healthcare professionals’ training curriculum could be hugely influential.

### Strengths and limitations

Medical registrars are key influencers of clinical debriefing in practice and so are a pertinent group to study. Lessons learnt from this group are relevant and transferable to a wide range of educators and organisations, as many teams will encounter the same barriers and enablers to CD. However, we recognise that some aspects of the medical registrar role are unique. This exact role may not be replicated in other healthcare systems, and, the clinical scenarios and acuity that they encounter may be different to that of other teams. Also, we focused on a single group within South East Scotland, and not all experiences will translate to other locations, which may limit the transferability of the results. A valuable avenue of future research would be to offer this method of training to interprofessional teams, as all members of a clinical team can be involved in CD.

All participants of the simulation course were invited to interview, and the participants were not pre-warned that the interview would explore CD. This reduced the potential self-selection of registrars with a strong interest in CD. However, we do acknowledge that participants’ attitudes and enjoyment of simulation may have influenced willingness to participate. This study was grounded in theory and Burke and Hutchins’ evaluation model [26] was highly relevant as a conceptual framework. However, we are mindful that it was developed within a human resources context and, similar to a previous study 27], we have applied it to a healthcare setting. This contextual shift means it is possible that other work environment influences exist within healthcare that were not emphasised by the chosen framework. On the other hand, the use of a pre-existing framework allows this study to build on others in a conceptually robust but practically grounded way. It also allows educators to transfer our research findings to their own context more easily.

### Future work

**Fig. 4:**
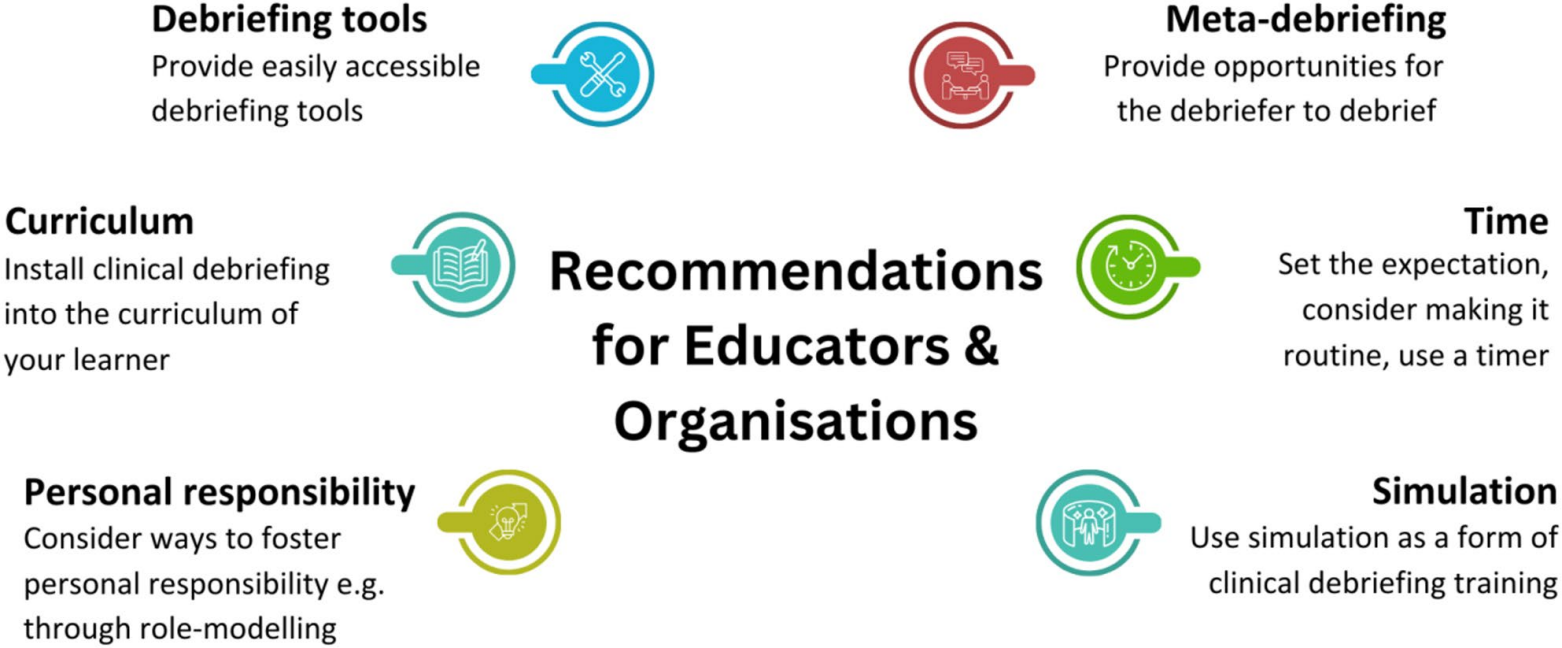
Recommendations for educators and organisations based on our findings

This study focused on the work environment, however it offers possibilities for future research investigating other aspects of transfer of learning, such as evaluating the simulation scenario itself. Whilst simulation appears to be useful training for CD and enhancing awareness of the practice, our findings indicate that in order to embed CD in healthcare organisations it must be linked closely with the work environment. With this in mind, educators and organisations must be cognisant of the transfer problem and design educational strategies – whether that be simulation or otherwise – that consider all aspects of training transfer. To this end, based on our findings, we have generated recommendations for educators and organisations hoping to incorporate CD into their own practice (Fig. 4).

## Conclusions

Recent literature explores the well-documented barriers to clinical debriefing; now is the time to catalyse widespread implementation. This study has explored the work environment barriers and enablers of CD, in relation to the transfer of learning from simulation to clinical practice. It has highlighted the importance of personal responsibility and workplace culture in implementing CD. This has moved our understanding from exploring the known barriers towards appreciating the enablers of CD, and has generated practical solutions to facilitate the update of CD in practice. We hope that organisations and educators will harness the learning generated through exploration of transfer from simulation to workplace practice, to promote the adoption of CD within their own setting. This will help to bridge the gap and make clinical debriefing the norm, not the exception.

## Supplementary Information

Supplementary Material 1.

Supplementary Material 2.

## Data Availability

Data is provided within the manuscript or the supplementary information file.

## Abbreviations

CD: Clinical debriefing
NHS: National Health Service
SBE: Simulation-based education
UK: United Kingdom
