# Supplementary material for: Transfer of clinical debriefing from simulation to practice: exploring the barriers and enablers
Source: Adv Simul (Lond). 2026 Jan 12;11:8. doi: 10.1186/s41077-025-00405-8 (PMC12895721; doi:10.1186/s41077-025-00405-8)
Supplement: Supplementary file 1 — Supplementary Material 1. [file 41077_2025_405_MOESM1_ESM.docx]

**Title: Med Reg Sim – Obstetric emergency / Clinical Event Debriefing**

**Participants:** Doctors working at medical registrar level (IMT 3 – 7)

**Estimated running time:** 20 mins

**Intended learning objectives:**

By the end of the simulated scenario and debrief, learners should be able to:

1. Demonstrate safe DC cardioversion in a pregnant patient
2. Facilitate a clinical event debriefing using the STOP5 tool
3. Understand that reflection around team dynamics and systems issues through clinical event debriefing can improve team performance and future patient care

**Case summary/ Story line:**

Agata Evans is a 30yr old female who is 34 weeks pregnant with her first pregnancy. She has no past medical history and isn’t on any medications. Her pregnancy has been uncomplicated so far apart from some acid reflux. She lives with her husband Mark and is usually fit and well.

Agata was admitted to the labour ward HDU due to palpitations and dizziness. She hasn’t had any symptoms since admission with a normal ECG, but has been kept in for a period of cardiac monitoring.

**Personnel:**

- Midwife (embedded faculty)
- Specialty registrar (obstetric or cardiology) (over phone)
- Anaesthetic registrar (embedded faculty)

**Faculty information:**

- 1 participant leading scenario, can allow 1-2 further participants to attend the scenario if prompted by the participant e.g. by a medical emergency
- Provide participants with brief pre-learning materials around clinical debriefing including STOP5
- Pre-brief – include in the main pre-brief at the start of the day that this will include a clinical event debrief within the scenario. Explain in the participant briefing immediately prior to the scenario that you would like the participant to facilitate a clinical debrief at the end of the scenario, and the tool (STOP5) for this will be provided
- Transition into clinical debrief – this should be prompted by embedded faculty if needed, and guide the participant into the designated area (screened off with chairs), which should include the STOP5 tool

**Faculty Scripts:**

**Obstetric Registrar** – in theatre with consultant at time of phone call, doing a section. Can answer questions over phone with scrub nurse holding to ear. Agrees to trial adenosine but can prompt to progress to DCCV if asked when patient deteriorates. Can prompt for uterine displacement. Will come as soon as possible but realistically will be 10-15mins. Will say can ask scrub nurse to alert theatre coordinator to be ready just in case.

**Anaesthetic Registrar –** agrees to attend if called. Can prompt to proceed to DCCV. Does not need to give sedation given GCS, supports airway

**Cardiology Registrar** – if called, gives advice that benefit / risk ratio in favour of control of arrythmia and management is the same as outwith pregnancy, including vagal maneuvers, adenosine, DCCV (with fetal monitoring and anaesthetic input).

**Embedded Faculty Input into Clinical Debrief:**

Midwife – offer manual displacement of uterus spontaneously in state 2

Points to bring up in STOP5:

- Midwife: You didn’t have adenosine in stock (mentioned by FY2) and had to get someone to run to A+E (before med reg arrived).
- Anaesthetic reg: The defib was unable to print off a rhythm strip
- Midwife: med reg number on whiteboard was wrong, so FY2 couldn’t get through (or switchboard wrong etc)

In addition: try to be authentic in your responses within the debrief based on what you witness within the scenario. You will hear a summary of the case and be asked to contribute on things that went well / opportunities to improve / action points. You could for example comment on what the communication was like (was there clear delegation of roles/tasks? Did everyone in the team feel that they knew what was going on?), whether there was a clear leader, any issues with equipment etc.

**Debrief Discussion points:**
Run a standard debrief after the scenario – aim to split into two parts. First half about the scenario itself (when the discussion will likely focus on DCCV in pregnancy – ILO1. Can offer a microteach if participants would like this) and the second half about CED (ILO 2/3).

**Briefing for candidate:**

This is the scenario with the clinical debrief, you will be asked to lead a short emergency and then facilitate a clinical debrief with the team within the room.

Scenario: You are the on call medical registrar at night in a district general hospital. You have been asked to review a patient in labour ward by the obstetric FY2 as they were concerned about a possible tachyarrhythmia. You will now hear the handover from the FY2 (voice note will play).

**Staff Nurse - Embedded professional**

- Hi, I’m the midwife looking after Agata. She suddenly said she was feeling funny and her pulse is through the roof...sorry, the FY2 has had to run off to help with an emergency section

| EVENTS | MANIKIN STATE | PARTICIPANT EXPECTED BEHAVIOUR | TRANSITION/ notes | PROMPT IF REQUIRED |
| --- | --- | --- | --- | --- |
| BASELINE  ILO 1:  DCCV | Patient response  “My heart is racing”  (no chest pain or dizziness at this point)  Physiology  BP 120/ 65, sats 94% RA, HR 200, RR 24  BM 7  Chest clear  ECG:  Active: narrow complex tachycardia rate 200 – 220  (waiting rhythm sinus rhythm rate 72 – for state 3)  Cannula in antecubital fossa  Fetal monitoring:  CTG on | A-E assessment  Investigations – ECG (available), bloods, ABG  Call for help early  Ask for / check protocols | Initial A-E completed  Escalation and advice (see faculty scripts)   - Obstetric reg - ICU / anaesthetic reg - Cardiology reg   May start treatment:   - Vagal manouvres - Trial adenosine bolus (no response) | Embedded professional:  Is there anything we can give her?  I think we might need some more help, should I put out a medical emergency call?  Patient:  ‘I feel a bit dizzy’  Physiology:  BP 100/60, HR 220, RR 28 |
| State 2  ILO 1:  DCCV | Other participants arrive to help  Patient  'I’m feeling really dizzy’  Physiology  BP 78/40  Sats 92% on air, 98% on O2.  HR 230  ECG unchanged  Patient then becomes unresponsive to pain  Arrival of anaesthetic reg as second embedded professional | Decision made to progress to DCCV  Expected to refer to anaesthetic reg for assistance with sedation - this is superseded by reduction in GCS  Midwife offers manual uterus displacement to the left at time of reduced GCS  [If calls for advice to cardio / consultant etc – agree need to progress to DCCV] | Successful DCCV on 2^nd^ shock (no need for sedation given low GCS and urgency)  Patient cardioverts, observations improved  ECG changed to  sinus rhythm rate 72  BP 124/76, HR 76, RR 22, sats 96% on RA  12 lead ECG SR rate 70 - 80 | Embedded professional (midwife)  She’s getting worse – what’s the plan here?  Embedded professional (anaesthetic reg):  What do you think about cardioversion? ALS guidelines say safe in pregnancy...  Physiology  HR 240, BP 62/34 |
| State 3  ILO 2/3  Clinical event debriefing | Obstetric registrar arrives and takes over “we will move her to labour ward, happy to take over”  Participant leading scenario to guide clinical debrief using STOP5 tool in screened off part of room or in another room  Faculty within sim scenario to take part in this and can raise systems problems if not raised by participants |  | Clinical event debriefing completed  NB embedded faculty – remember to turn off defib and lead team to screened off area or debrief room | Prompt by facilitator / from pre-brief  “she’s stable now, shall we have a quick debrief”  Move to screened off area of the sim ward  CED should last no longer than 5 mins |

| Equipment required |  |
| --- | --- |
| Equipment: | **Where to acquire:** |
| Manual defibrillator | Sim suite |
| Bedside cabinet | Sim suite |
| Drip stand | Sim suite |
| Simman – obstetric | Sim suite |
| Monitoring equipment | Standard in Sim suite |
| Oxygen options – non rebreath face mask, venturi + adapters, nasal cannulae | Sim suite |
| Left lateral tilt resources (towels or wedge) |  |
| Equipment for IV access, bloods, ABG | Sim suite |
| BNF | Sim suite |
| Fluids (plasmalyte, 0.9% saline) + giving set | Sim store room |
| Drugs: adenosine | Drug cabinet |
| Obstetric ALS guidance notes, narrow complex tachycardia ALS guidance | Scenario folder |
| A+E notes | Scenario folder |
| ECG 1 (sinus rhythm), ECG 2 (narrow complex tachycardia rate 220). | Scenario folder or electronic resources |
| CTG | Scenario folder + simulated CTG machine (box) |
| Kardex | Scenario folder |
| MEWS (modified early obstetric warning score) | Scenario folder |

| **Author(s)** | Kat Ralston |
| --- | --- |
| **Contributor(s)** | Alex Stirzaker, Janey McKane, Vicky Tallentire, Jo Kerins, Emma Phillips, Ed Mellanby, Gillian Fordyce |

| **Version** | **Effective From** | **Effective To** | **Change Summary** |
| --- | --- | --- | --- |
| 1.1 | 15.11.22 | 01.04.23 | Updated to be a FY2 referral initially (not a medical emergency). Prompt to turn off defib before STOP5. Faculty to prompt STOP5 |
| 1.2 | 01.04.23 | 3.7.23 | Updated to include prompts for ‘system issues’ for embedded faculty for CED |
| 1.3 | 16.09.24 | - | Recorded FY2 handover on voice note, updated briefing for candidate, will print faculty information on a separate page |
