# Supplementary material for: Transfer of clinical debriefing from simulation to practice: exploring the barriers and enablers
Source: Adv Simul (Lond). 2026 Jan 12;11:8. doi: 10.1186/s41077-025-00405-8 (PMC12895721; doi:10.1186/s41077-025-00405-8)
Supplement: Supplementary file 2 — Supplementary Material 2. [file 41077_2025_405_MOESM2_ESM.docx]

**Additional File 2**

Interview schedule from the study exploring the work environment enablers and barriers to clinical debriefing

**Context & Registrar Characteristics**

1. Purpose of interview
   1. Clarify definition of clinical debriefing
      1. the attempt to bridge the gap between experience of an event and making sense of it
2. Check happy with consent (completed at time of simulation course). Highlight option to withdraw at any time without giving a reason until data analysis
3. Confirm happy for recording and explain anonymity process
4. Trigger warning. Option to pause / stop. Signpost to ability to discuss with researcher / peer support if needed
5. Explain need to avoid any patient or colleague identifiable details (and names in interview)
6. Demographics (check provided this within consent form – if not, prompt to do so)

**Opening Questions**

1. Do you think clinical debriefing is important? If so – why? Or why not?
2. What do you think is the purpose of clinical debriefing?
3. What do you think are the enablers and barriers to clinical debriefing?

**Training Design**

1. Do you have any experiences of clinical debriefing before the simulation? What happened?
2. What do you remember about the specific simulation scenario that incorporated the clinical debriefing?
   1. Did you learn anything from the scenario? If so, what? Otherwise, why not?
   2. Do you think the scenario has changed your ‘real-life’ experience of clinical debriefing? If so in what way?
   3. How has the training affected your thoughts on clinical debriefing, its challenges, your perception of it and whether you could do it in the real world?

**Work Environment**

1. Can you think of a situation since the simulation where a clinical debriefing might have been useful or appropriate?
   1. Was it undertaken?
   2. If yes:
      1. Tell me more about that? What happened (setting/event/type of debrief)? What helped you to take that step? How did it go? Have you / would you do it again?
      2. Any other times?
   3. If no:
      1. What stopped the debriefing from happening?
      2. Do you think there were other unspoken factors influencing whether it did or didn’t happen?
      3. What do you think could have made it happen? What could you have done to make it happen? Did you feel able to do that? Why or why not?
      4. Any other times?
2. Have you ever been given a clinical debriefing tool? Would this help?
3. Does it make a difference if you’re in a stable or *ad hoc* team (which varies with each shift)? Would that make it more or less likely to happen?
4. Who is responsible for initiating and/or leading a clinical debriefing?
   1. To what extent do you feel obliged to debrief?
   2. Who’s accountable – who is responsible to ensure you do it? At what level does that responsibility come from?
   3. If it was mandated would you do it?
   4. Is there someone to debrief you? What difference might that make?
5. Is there a supportive culture towards clinical debriefing in your area of work?
   1. Do your supervisors and peers encourage clinical debriefing?
   2. Does the department or organisation you’re working for support clinical debriefing?

**Close of interview**

1. Is there anything else that we haven’t discussed that you would like to share?
